# Surgeon Skill and Perioperative Outcomes in Robot-Assisted Partial Nephrectomy

**DOI:** 10.1001/jamanetworkopen.2024.21696

**Published:** 2024-07-15

**Authors:** Yuzhi Wang, Samantha Wilder, Mahmoud Hijazi, Marquisha D. Myles, Mahin Mirza, Monica Van Til, Thomas Maatman, Khurshid R. Ghani, Brian R. Lane, Craig G. Rogers

**Affiliations:** 1 Vattikuti Urology Institute, Henry Ford Health, Detroit, Michigan; 2 Department of Urology, University of Michigan Medical School, Ann Arbor; 3 Michigan Urological Clinic, Grand Rapids; 4 Corewell Health Hospital System, Grand Rapids, Michigan; 5 Michigan State University College of Human Medicine, Grand Rapids

## Abstract

**Importance:**

Technical skill in complex surgical procedures may affect clinical outcomes, and there is growing interest in understanding the clinical implications of surgeon proficiency levels.

**Objectives:**

To determine whether surgeon scores representing technical skills of robot-assisted kidney surgery are associated with patient outcomes.

**Design, Setting, and Participants:**

This quality improvement study included 10 urological surgeons participating in a surgical collaborative in Michigan from July 2021 to September 2022. Each surgeon submitted up to 7 videos of themselves performing robot-assisted partial nephrectomy. Videos were segmented into 6 key steps, yielding 127 video clips for analysis. Each video clip was deidentified and distributed to at least 3 of the 24 blinded peer surgeons from the collaborative who also perform robot-assisted partial nephrectomy. Reviewers rated technical skill and provided written feedback. Statistical analysis was performed from May 2023 to January 2024.

**Main Outcomes and Measures:**

Reviewers scored each video clip using a validated instrument to assess technical skill for partial nephrectomy on a scale of 1 to 5 (higher scores indicating greater skill). For all submitting surgeons, outcomes from a clinical registry were assessed for length of stay (LOS) greater than 3 days, estimated blood loss (EBL) greater than 500 mL, warm ischemia time (WIT) greater than 30 minutes, positive surgical margin (PSM), 30-day emergency department (ED) visits, and 30-day readmission.

**Results:**

Among the 27 unique surgeons who participated in this study as reviewers and/or individuals performing the procedures, 3 (11%) were female, and the median age was 47 (IQR, 39-52) years. Risk-adjusted outcomes were associated with scores representing surgeon skills. The overall performance score ranged from 3.5 to 4.7 points with a mean (SD) of 4.1 (0.4) points. Greater skill was correlated with significantly lower rates of LOS greater than 3 days (−6.8% [95% CI, −8.3% to −5.2%]), EBL greater than 500 mL (−2.6% [95% CI, −3.0% to −2.1%]), PSM (−8.2% [95% CI, −9.2% to −7.2%]), ED visits (−3.9% [95% CI, −5.0% to −2.8%]), and readmissions (−5.7% [95% CI, −6.9% to −4.6%]) (*P* < .001 for all). Higher overall score was also associated with higher partial nephrectomy volume (β coefficient, 11.4 [95% CI, 10.0-12.7]; *P* < .001).

**Conclusions and Relevance:**

In this quality improvement study on video-based evaluation of robot-assisted partial nephrectomy, higher technical skill was associated with lower rates of adverse clinical outcomes. These findings suggest that video-based evaluation plays a role in assessing surgical skill and can be used in quality improvement initiatives to improve patient care.

## Key Points

**Question:**

Is surgeon technical skill associated with perioperative outcomes for robot-assisted partial nephrectomy?

**Findings:**

In this quality improvement study that included peer assessments of surgical skill for 10 urological surgeons, higher technical skill was associated with lower rates of extended hospital stay, high estimated blood loss, positive surgical margins, 30-day emergency department visits, and 30-day readmissions. Higher annual partial nephrectomy volume was associated with higher technical skill.

**Meaning:**

These findings suggest that video-based evaluation plays a beneficial role in assessing surgical skill and should be considered in quality improvement initiatives to improve patient care.

## Teaser

This quality improvement study assesses whether surgeon scores representing technical skills of robot-assisted kidney surgery are associated with patient outcomes.

## Introduction

Surgical technical skills may be variable between surgeons and may influence intraoperative and postoperative outcomes.^1^ Variability in surgeon factors such as fellowship, experience, and surgical approach may also impact outcomes.^2^ Video-based evaluation has been used across different surgical fields to evaluate proficiency and has been useful in examining variability in technique, outcomes, and areas of improvement for surgeons and trainees.^3,4,5,6^ For example, video review for bariatric surgeons demonstrated that better surgical skills were associated with lower 30-day postoperative complications, reoperation, readmission, and visits to emergency departments.^7^ Similar studies with laparoscopic sleeve gastrectomy and pancreaticoduodenectomy have demonstrated an association between skill and outcomes.^8,9^ In the setting of oncologic surgery, the data are limited, with only 1 study demonstrating that better technical performance after laparoscopic gastric cancer surgery predicted lower postoperative complications.^10^ However, to our knowledge, no studies have assessed the association of skill with oncologic outcomes such as the completeness of tumor resection with negative surgical margins.

For localized kidney cancer, partial nephrectomy is the standard of care and is generally preferred over radical nephrectomy when possible.^11^ The utilization of robot-assisted partial nephrectomy has been trending upwards.^12,13,14^ Between 2009 and 2012, the proportion of robot-assisted partial nephrectomies increased from 20% to 49%, with recent literature reporting greater than 60%.^13,15^ While robot-assisted partial nephrectomy is commonly performed, it is considered a technically challenging surgery, and potential complications include hemorrhage, urinary leakage, the need to convert to radical nephrectomy and/or open surgery, the failure to entirely remove cancer, and other general postoperative complications.^16^ These events can have profound implications for health care expenditures, especially if patients require hospital readmission, reintervention, and/or additional therapies for cancer control. To date, there have been no studies that have assessed the technical quality of surgeons performing this surgery and its association with patient outcomes.

For these reasons, we conducted a video-based evaluation to determine whether surgical skill in robot-assisted partial nephrectomy was associated with perioperative outcomes, including immediate oncologic control. We hypothesized that surgeons with greater technical skill would have lower rates of adverse events and improved oncologic outcomes. Our work aims to inform surgeons, patients, and stakeholders such as payers, professional societies, and hospital systems, because if skill is associated with outcomes, it would suggest that interventions to measure and improve surgical skill would be of benefit to the wider community.

## Methods

### Study Overview

This study was conducted within the Michigan Urological Surgery Improvement Collaborative (MUSIC), a consortium of hospitals and urologic surgeons aiming to improve the quality of urologic care in Michigan funded by Blue Cross Blue Shield of Michigan. MUSIC is composed of 46 diverse practices with more than 260 urologists from those facilities. MUSIC-Kidney Mass: Identifying and Defining Necessary Evaluation and Therapy (KIDNEY) is a division with a focus on treatment of kidney cancer and consists of 20 practices and more than 150 urologists. MUSIC-KIDNEY maintains a prospective clinical registry of all newly diagnosed localized kidney masses up to 7 cm (≤stage T1) in participating practices. Details on the design, inception, and data collection have been published previously.^17^ Trained data abstractors at each clinical site review primary medical records and enter clinical data into a web-based registry. Treatment and follow-up data are recorded at set intervals, and data are periodically audited for quality control. Each practice in MUSIC obtained exemption or approval for collaborative participation from a local institutional review board . Given the quality improvement focus of MUSIC, the initiative was deemed exempt from review. Participant consent was waived because the institutional review board was exempt for quality improvement initiatives. This quality improvement study has been prepared in accordance with the revised Standards for Quality Improvement Reporting Excellence (SQUIRE) reporting guideline version 2.0.^18^

The primary objective of this study was to assess the association between surgeon technical skills and perioperative outcomes. Secondary objectives were to assess the distribution of technical skill within our participating surgeons, and the association of surgical volume with scores and outcomes. Lastly, we also wanted to evaluate levels of participant satisfaction.

### Video Submission From Surgeons

We invited surgeons in the collaborative to submit at least 1 video of themselves performing robot-assisted partial nephrectomy for a patient with a cT1 kidney mass. Surgeons were asked to submit a recent and representative case and the ultimate choice was left to their discretion. Videos were deidentified prior to submission.

Initially 14 surgeons volunteered and submitted 35 videos to the MUSIC coordinating center from July 2021 to August 2022. We prepared 28 videos from 11 surgeons for review, editing them into video clips of 6 critical components of the procedure: exposure of the kidney, identification of the ureter and gonadal vessels, hilar dissection, tumor localization and exposure, clamping and tumor resection, and renorrhaphy. In total, there were 127 video clips; not all submitted videos contained all 6 steps. Video clips were randomized and electronically distributed to peer surgeon reviewers.

### Measures of Technical Skill

We evaluated technical skills using a recently published and internally validated tool called Scoring for Partial Nephrectomy (SPaN), which is used specifically for robot-assisted partial nephrectomy. Each of the 6 domains was scored on a 5-point Likert scale (1 = lowest to 5 = highest score). Specific scoring criteria for each step were outlined by Iqbal et al.^19^

### Video Review Process

All surgeons in the collaborative familiar with performing robot-assisted partial nephrectomy were invited to participate as peer reviewers. There were 24 surgeons from 14 MUSIC practices who volunteered as peer reviewers. Blinded reviewers were sent a set of randomized videos clips to score. Reviewers were given a score sheet with descriptions of the scoring criteria; reviewers scored videos objectively and provided subjective written feedback in a free-text box. Each video clip was evaluated by at least 3 different surgeons. This resulted in a total of 383 reviews. The overall process is summarized in eFigure 1 in Supplement 1 and occurred between August 2022 and March 2023. All reviewers reviewed a different subset of videos. Eight out of the 11 surgeons who submitted videos were also peer reviewers. Reviewers who were also submitters were not sent any of their own video clips. There were 27 different participants overall.

An individualized surgeon score card was sent to participating surgeons after completion of video review (eFigure 2 in Supplement 1). The report contained specific scores pertaining to the individual. Surgeons also received free-text comments from reviewers and a link to the video clips. A survey was sent out to all participants as well to assess level of satisfaction and efficacy of the video review (eFigure 3 in Supplement 1). Submitters and reviewers received different surveys where the survey wording was slightly different for submitters and reviewers. The survey questions utilized responses on a Likert scale from 1 to 5 (1 = highly disagree, 2 = disagree, 3 = neutral, 4 = agree, 5 = highly agree).

### Outcomes

Outcomes from the MUSIC registry were obtained for all MUSIC-KIDNEY surgeons with at least 5 cases of partial nephrectomy, including the submitting surgeons. Outcome measures included length of stay (LOS) greater than 3 days, estimated blood loss (EBL) greater than 500 mL, warm ischemia time (WIT) greater than 30 minutes, positive surgical margins (PSM), emergency department (ED) visits, and readmissions within 30 days postoperatively.

### Statistical Analysis

The statistical analysis regarding perioperative outcomes included 10 surgeons as 1 surgeon had only 1 partial nephrectomy entered into the registry and was thereby excluded. Additionally, analysis only included surgeons with 5 or more cases with data for each end point; 1 surgeon each had incomplete data for WIT and LOS.

The overall performance score was used as the summary measure of technical skill for each surgeon, calculated as an average of the mean scores across the 6 domains. Cronbach α of 0.72 indicated an acceptable level of internal consistency between scores of each domain to justify the use of an overall score. Mean score for each domain was calculated by averaging all scores received for video clips specific to each domain.

Each video clip was reviewed by a different subset of reviewers, which introduced the potential for rater bias. To identify potentially biased raters, we calculated *z* scores for each peer reviewer. For each video clip, a *z* score was calculated as the difference in score of each reviewer from the average score of all the reviewers across that domain, divided by the SD. No peer reviewer’s *z* score was significantly different from the average. Therefore, the main analysis was not adjusted for interrater variability.

We then evaluated the association between overall technical skill scores and each outcome of interest. First, a patient-level mixed-effects logistic regression model for each outcome with patients nested within each surgeon, was used to adjust for confounding factors and obtain risk-adjusted rates of outcomes for each surgeon. Adjustment variables used in each model included patient age, patient gender, Charlson comorbidity index, tumor size, tumor type, and RENAL (radius, exophytic/endophytic, nearness hilum, anterior/posterior, location relative to polar lines) nephrometry score. Insurance type was also included as a variable in the risk-adjusted model for rates of ED visits and readmissions. Only age and tumor size were continuous variables; the remaining variables were categorical. This process used robot-assisted partial nephrectomy data from all MUSIC-KIDNEY urologists to obtain a more precise risk adjustment. Then, a simple linear regression was used to test the association between the overall skill scores with the predicted probabilities of the outcomes. A β coefficient was generated for each linear regression and is a measure of the slope of the line of best fit; the β coefficient indicates the change in rate of an outcome associated with a 1-point increase in score. If the overall skill score was significantly associated with the outcome, then the analysis was repeated for each of the key steps.

We also evaluated the association between overall technical skill scores and surgeon volume using the same aforementioned method. Surgeon volume was defined as the annual number of robot-assisted partial nephrectomies completed. Although it is beyond the scope of our paper, we also evaluated the presence of trainees through telestrations, and did not find a clinically important difference in outcomes.

All analyses were performed using SAS version 9.4 (SAS Institute) from May 2023 to January 2024. Historically, MUSIC-KIDNEY set statistical significance at *P* = .05. To adjust for multiple comparisons, the Bonferroni correction was used to modify the statistical significance level. In this manuscript, statistical significance was set at *P* = .008 (0.05 / 6 comparisons) for testing the overall scores with each of the outcomes and for testing the scores from each of the 6 steps with the outcomes.

## Results

**Figure 1.**
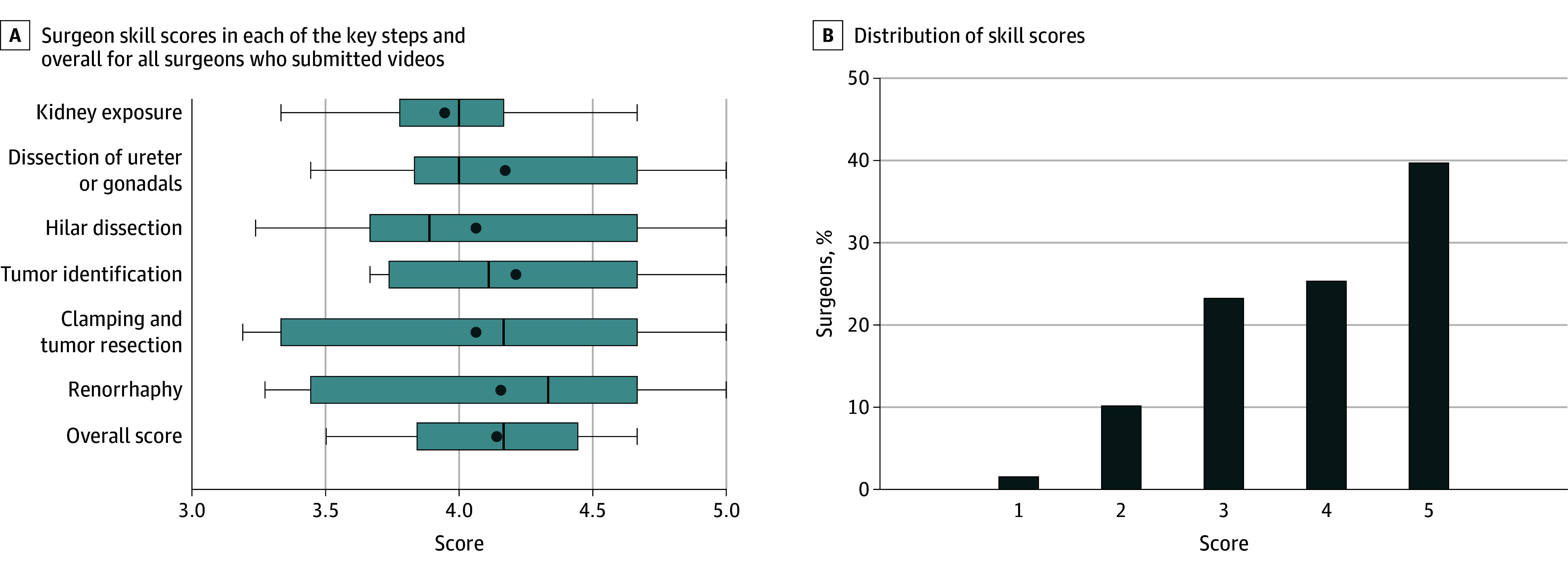
Surgeon Skill Scores A, Boxplots describing the distribution of overall surgeon skill scores in each of the 6 key steps for all surgeons who submitted videos. The dot represents the mean overall score, and the vertical line inside the box represents the median overall score among all submitting surgeons. The box represents the IQR, and the whiskers of the boxplot represent the range of overall scores. B, Bar chart showing distribution of scores given in the 383 reviews. The x-axis numbers (1-5) refer to the numerical scoring of surgical skill per video clip.

A total of 27 unique surgeons participated in this study; the median (IQR) age was 47 (39-52) years; 24 participants (89%) were male and 3 (11%) were female. The overall performance score representing technical skill ranged from 3.5 to 4.7, with a mean (SD) of 4.1 (0.4). Mean scores across the 6 key steps, represented by the vertical line inside each boxplot in Figure 1A, ranged from 3.9 to 4.2 with SDs ranging from 0.43 to 0.69. Out of 383 reviews, 6 reviews (1.6%) received a score of 1, 39 (10%) received a 2, 89 (23%) received a 3, 97 (25%) received a 4, and 152 (40%) received a 5 (Figure 1B). The Table describes the patient and tumor characteristics for patients who underwent robot-assisted partial nephrectomy completed by the participating surgeons who submitted videos.

**Table.** Characteristics of Patients With T1 Kidney Mass Who Underwent Robot-Assisted Partial Nephrectomy Completed by Surgeons Who Submitted Videos

Variable	No. (%) (N = 569)
Age, median (IQR), y	60 (50-68)
Sex	
Male	342 (60)
Female	227 (40)
Race	
African American	51 (9.0)
American Indian or Alaska Native	4 (0.7)
Asian	6 (1.1)
White	434 (76)
Unknown	61 (11)
Other^a^	13 (2.3)
Charlson comorbidity index	
0	335 (59)
1	108 (19)
≥2	126 (22)
Tumor size, median (IQR), cm	3.0 (2.2-3.9)
Tumor type	
Solid	469 (82)
Complex cyst	48 (8.4)
Indeterminate	42 (7.4)
Other	10 (1.8)
RENAL nephrometry score^b^	
Low (4-6)	166 (43)
Intermediate (7-9)	189 (49)
High (10-12)	33 (8.5)
Insurance type^b^	
Private	361 (64)
Public	199 (35)
None	8 (1.4)

Abbreviation: Renal indicates radius, exophytic/endophytic, nearness hilum, anterior/posterior, location relative to polar lines.

^a^ Other race includes patients who were not in any of the 5 listed categories.

^b^ Data not available for insurance type (n = 1) and RENAL nephrometry score (n = 181).

**Figure 2.**
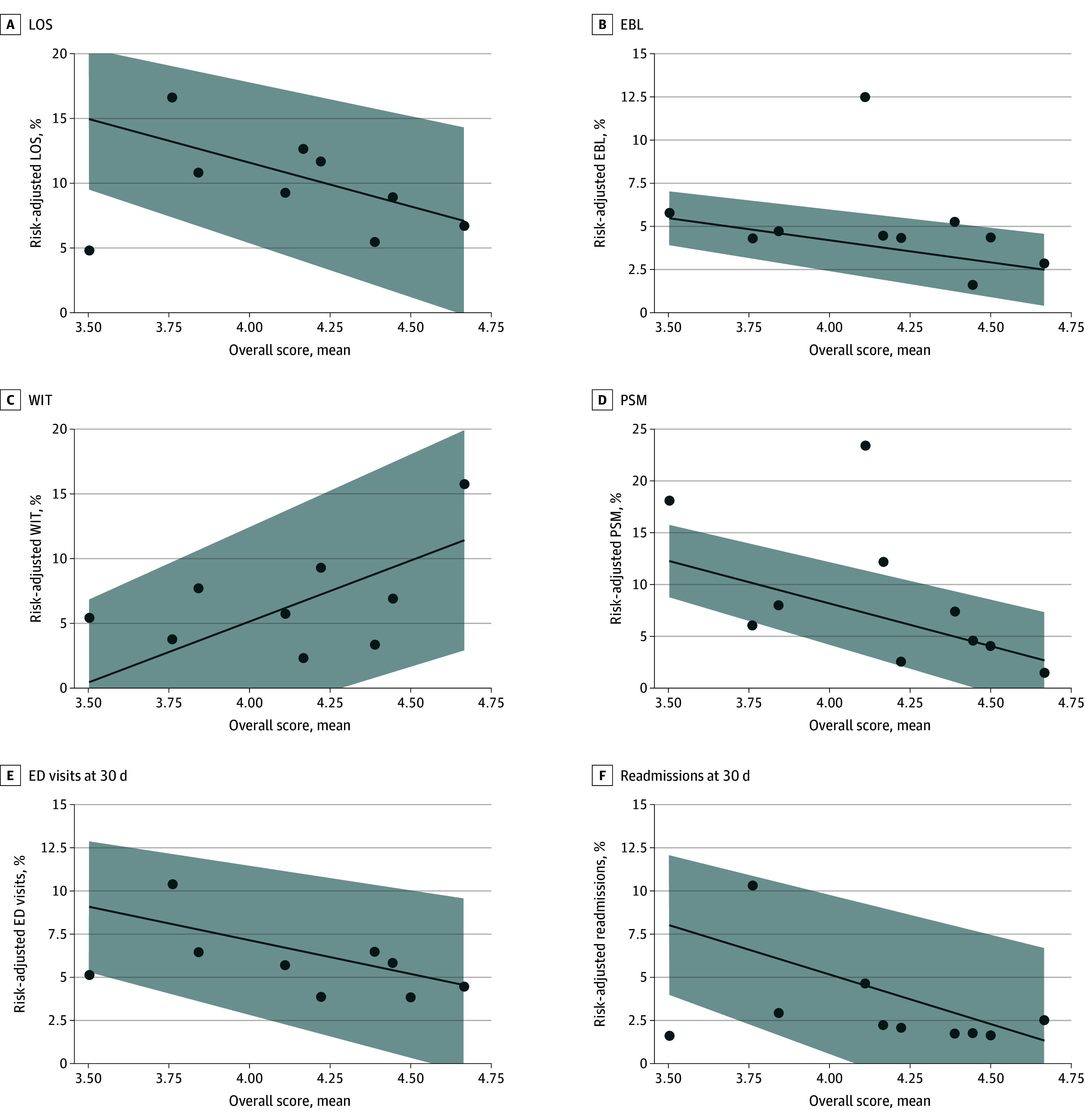
Surgeon Scores on Individual Videos and Rate of Risk-Adjusted Outcomes for a Surgeon’s Panel of Partial Nephrectomies Each dot in the scatterplot represents an individual urologic surgeon. Line of best fit and the 95% CI (shaded area) are shown. The β coefficient is a measure of the slope of the line of best fit, which indicates the change in rate of an outcome associated with a 1-point increase in score. Scores were significantly associated with all outcomes: length of stay (β = −6.8%; 95% CI, −8.3% to −5.2%), estimated blood loss (β = −2.6% [95% CI, −3.0% to −2.1%), warm ischemia time (β = 9.4%; 95% CI, 7.6% to 11.0%), positive surgical margins (β, −8.2%; 95% CI, −9.2% to −7.2%), 30-day emergency department visits (β = −3.9%; 95% CI, −5.0% to −2.8%), and 30-day readmissions (β = −5.7%; 95% CI, −6.9% to −4.6%) (all *P* < .001). EBL indicates estimated blood loss; ED, emergency department; LOS, length of stay; PSM, positive surgical margin; WIT, warm ischemia time.

There were significant associations between overall surgeon technical skill and perioperative outcomes. On average, 1 point higher in overall score was significantly associated with a 6.8% lower rate (95% CI, −8.3% to −5.2%) of prolonged hospital LOS, a 2.6% lower rate (95% CI, −3.0% to −2.1%) of extensive EBL, a 9.4% higher rate (95% CI, 7.6% to 11%) of prolonged WIT, an 8.2% lower rate (95% CI, −9.2% to −7.2%) of PSM, a 3.9% lower rate (95% CI, −5.0% to −2.8%) of 30-day ED visits , and a 5.7% lower rate (95% CI, −6.9% to −4.6%) of 30-day readmissions (*P* < .001 for all outcomes) (Figure 2). Additionally, surgeon performance in several, if not all, key steps were significantly associated with outcomes (eTable 1 in Supplement 1). Higher technical skill in the hilar dissection, clamping and tumor resection, and renorrhaphy steps were significantly associated with lower rates of extensive EBL (−2.1% [95% CI, −2.4% to −1.8%] for hilar dissection step, −1.4% [95% CI, −1.7% to −1.1%] for clamping and tumor resection step, and −2.4% [95% CI, −2.8% to −2.1%] for renorrhaphy step; *P* < .001 for all). Higher technical skill in clamping and tumor resection and renorrhaphy were significantly associated with higher rates of prolonged WIT (1.4% [95% CI, 0.6% to 2.1%] for clamping and tumor resection and 3.7% [95% CI 2.1% to 5.4%] for renorrhaphy; *P* < .001 for both). Notably, higher skill in clamping and tumor resection was significantly associated with a mean 3.5% lower rate (95% CI, −4.2% to −2.9%; *P* < .001) of PSM.

**Figure 3.**
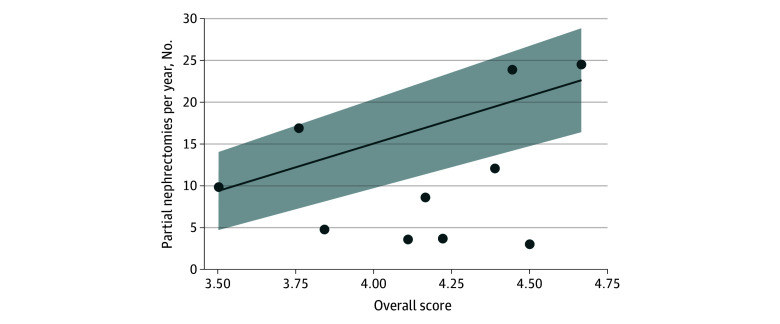
Overall Surgeon Skill Scores and Annual Partial Nephrectomy Volume Each dot in the scatter plot represents 1 of 10 urologic surgeons. Line of best fit and the 95% CI (shaded area) are shown. β coefficient (slope) was 11.4 (95% CI, 10.0-12.7) (*P* < .001).

Median (IQR) annual partial nephrectomy volume was 9 (4-17) nephrectomies. Higher annual partial nephrectomy volume was significantly associated with a better overall score: 1 point higher in overall skill score was associated with a mean of 11 (95% CI, 10 to 13) additional partial nephrectomies per year (*P* < .001) (Figure 3). Specifically, annual partial nephrectomy volume was significantly associated with technical skill in all key steps of the surgery except for identification of the ureter and gonadal vessels (eTable 2 in Supplement 1).

**Figure 4.**
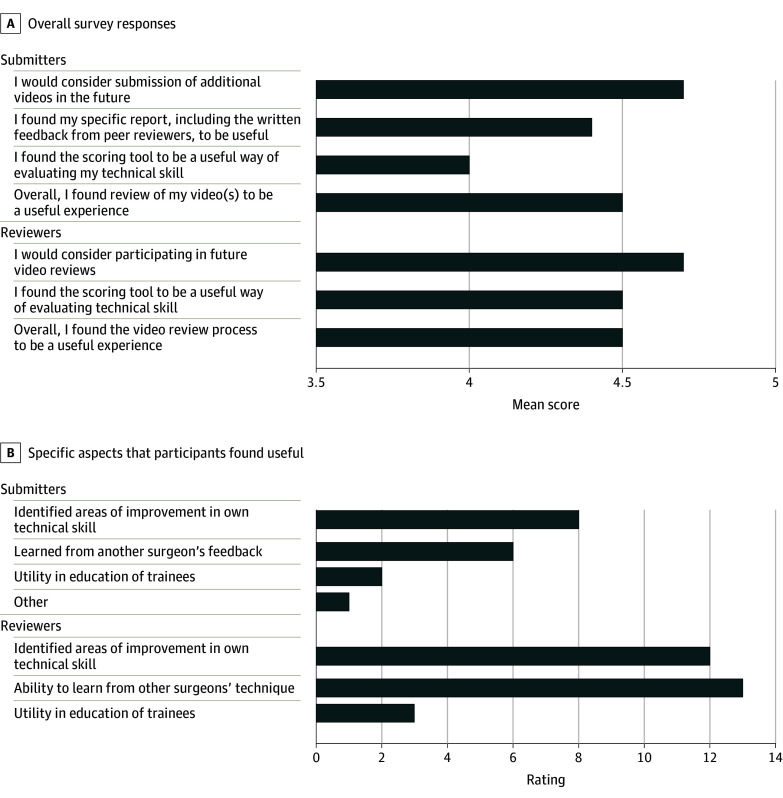
Survey Results to Assess Efficacy and Participant Satisfaction of the Video Review Process The survey was administered to all participants 2 weeks after receiving their score card. It used a Likert scale where 1 = highly disagree, 2 = disagree, 3 = neutral, 4 = agree, 5 = highly agree. Responses are labeled for 2 categories: (1) submitting surgeons and (2) peer reviewer surgeons. A, Average response from 15 participants. B, Specific aspects of video review that participants found useful. Indicated are the responses; one submitter selected “other” and specified “interesting to hear other perspectives.”

Fifteen of 27 participants (56%) filled out the postvideo review survey. Two were video submitters only, 5 were reviewers only, and 8 were both submitters and reviewers. Overall, the survey results showed high participant satisfaction. Participants especially valued the ability to learn from other surgeons’ techniques, to identify areas for improvement in their own technical skill, and to utilize the feedback for education of trainees (Figure 4). One urologist thought that the “inclusion of comments in addition to score was very helpful.” Another reviewer pointed out that “there were things [they] liked while watching, others [they] did not like, but in general it was very valuable to see different techniques.” Another submitter critiqued the heterogeneity of the reviewer feedback, saying “it was hard to interpret discordant comments, but it overall is still beneficial” (Figure 4).

## Discussion

In our study, urologic surgeons in Michigan were evaluated on their technical skills for robot-assisted partial nephrectomy, a common yet complicated surgery. To the best of our knowledge, this is the first study to assess technical skills in each major step of a robot-assisted oncologic procedure and show an association of skills with clinical and oncologic outcomes. We reported several notable associations: overall higher technical skill was significantly associated with lower rates of hospital LOS, extensive EBL, PSM, 30-day ED visits, and 30-day readmissions. Some steps do not appear directly associated with certain outcomes; for example, how well a surgeon dissects the kidney from neighboring organs may not directly affect surgical margin results on final pathology. However, we believe that these associations may serve as an indicator of lower or higher technical skill overall.

Other prior studies have described predictive patient and surgical factors that may affect perioperative outcomes. Greater surgeon experience was associated with lower operative time, WIT, and major postoperative complications for robot-assisted partial nephrectomy. However, body mass index, patient age, and kidney complexity also had significant associations with the aforementioned outcomes.^20^ Enucleoresection was associated with a significantly higher risk of PSM compared with resection (odds ratio [OR], 5.73; *P* = .02) and enucleation (OR, 2.42; *P* = .03).^21^ Laparoscopic partial nephrectomy was associated with a significantly higher risk of PSM compared with the open approach (OR, 1.36; *P* = .02).^22^

There was a significant association between technical skills and surgical volume. This is important as previous studies have found an association between surgical volume and perioperative outcomes. In a prior MUSIC-KIDNEY study, multivariable analysis showed a significant association between annual practice volume and PSM (OR, 0.96 [95% CI, 0.94-0.98]; *P* < .001).^23^ A National Cancer Database study from Xia et al^24^ reported that a higher hospital volume was associated with a lower odds of PSM, prolonged LOS greater than 3 days, and conversion from robot-assisted to open partial nephrectomy.

To our knowledge, our study is the first to display the complex interplay between surgical volume, technical skills, and various perioperative outcomes. Our study adds to multiple studies that found higher surgical case volume to be associated with improved outcomes; however, we are the first to use an objective measure of surgical skill.^24,25,26^ Our findings are not meant to discourage lower-volume surgeons, but rather to provide targets and specific areas for improvement for future quality improvement initiatives. One surprising finding was that higher surgeon skill score was associated with higher rate of prolonged WIT (9.4% per 1 point), perhaps because the higher scoring and more experienced surgeons get referred difficult cases that require a longer clamp time. Another surprising finding was that higher surgeon skill score in step 1, exposure of the kidney, was associated with higher rates of undesirable outcomes. This perhaps may be attributed to increased trainee involvement in the initial steps of partial nephrectomy, or skilled surgeons spending less time on step 1 while focusing on the more important later parts of the procedure.

Our survey results from participants were generally positive and suggest that video-based evaluation may serve purposes well beyond the scope of research studies. The idea of coaching and self-reflection through video review is a common tactic employed in sports. Now, video-based evaluation has been gaining traction in the surgical community, which has realized the value of video review and investigated coaching initiatives to improve surgical skills.^27,28,29,30^ After the conclusion of our video review, we developed a YouTube site housing the videos and their respective feedback from this study (eFigure 4 in Supplement 1). We hope this resource will be helpful for surgeons who want to learn new robotic partial nephrectomy techniques and improve their own abilities through online videos.

### Limitations

We recognize that there are limitations to our study. Only 27 surgeons participated as a submitter and/or reviewer, which is a subset of MUSIC surgeons who perform robot-assisted partial nephrectomy. Therefore, other different surgical techniques and opinions may not be represented by our data and outcomes. The scoring system used to assess surgeon skill does not account for certain technical variations, patient comorbidities, and procedural complexity.^19^ While all reviewers received a score sheet that delineates the scoring rubric and criteria, it is possible that a surgeon’s subjective opinion of the video clip affected the objective score given. However, analysis of interrater variability did not reveal any significant outliers that would require adjustment of scores. Submission of videos for review was voluntary; self-selection by the surgeon may have right skewed the distribution of scores. Patient and tumor characteristics, as well as postoperative pathology, were not provided with the submitted videos to prevent identification of patients; as a result, reviewers were not able to integrate the information that impacts surgical decision-making into their scoring thought process. We were therefore not able to directly correlate certain findings in the videos to scores and postoperative outcomes.

In light of the limitations, our findings still have direct implications to a multitude of stakeholders including patients, physicians, hospitals, and payers. Surgical complications continue to be a substantial economic burden in the health care system.^31,32,33^ Incomplete cancer resection and readmissions are notable outcomes when considering health care utilization.^32^ In the setting of kidney cancer, a positive margin is associated with higher recurrence and metastasis rates. The American Urological Association kidney cancer guidelines recommends more frequent surveillance if a patient has positive surgical margins at partial nephrectomy.^34^ Therefore, having a positive margin would lead these patients to have more frequent surveillance, additional treatment, and/or other burdens associated with disease progression.^35^ When extrapolating our results on perioperative outcomes, higher rates of PSM may lead to long-term poorer oncologic control. Readmission postoperatively is an undesirable outcome that incurs cost for the patient and for the hospital. Therefore, any intervention that may decrease PSM rates and readmissions, such as improving surgical proficiency, is worth pursuing.

## Conclusions

Using video review of robot-assisted partial nephrectomy, this quality improvement study found that technical skill was significantly associated with better outcomes. This evaluation modality may help surgeons improve their proficiency, which has implications toward better patient care and oncologic control.

## Notes

Supplement 1. **eFigure 1.** Outline of the Video Review Process **eFigure 2.** Example of a Score Card Given to Participants **eFigure 3.** Survey Sent to Submitters and Reviewers to Evaluate Satisfaction and Efficacy of the Video Review Process **eFigure 4.** The MUSIC-KIDNEY Urology YouTube Channel Contains Shortened Videos of All 127 Submitted Video Clips **eTable 1.** The Association Between Surgeon Skill Scores and Rate of Risk Adjusted Outcomes (%) in Each of the Six Key Steps **eTable 2.** The Association Between Surgeon Skill Scores and Annual Partial Nephrectomy Volume in Each of the Six Key Steps

Supplement 2.
Data Sharing Statement
