# Supplementary material for: Surgeon Skill and Perioperative Outcomes in Robot-Assisted Partial Nephrectomy
Source: JAMA Netw Open. 2024 Jul 15;7(7):e2421696. doi: 10.1001/jamanetworkopen.2024.21696 (PMC11250260; doi:10.1001/jamanetworkopen.2024.21696)
Supplement: Supplement 1. — eFigure 1. Outline of the Video Review Process eFigure 2. Example of a Score Card Given to Participants eFigure 3. Survey Sent to Submitters and Reviewers to Evaluate Satisfaction and Efficacy of the Video Review Process eFigure 4. The MUSIC-KIDNEY Urology YouTube Channel Contains Shortened Videos of All 127 Submitted Video Clips eTable 1. The Association Between Surgeon Skill Scores and Rate of Risk Adjusted Outcomes (%) in Each of the Six Key Steps eTable 2. The Association Between Surgeon Skill Scores and Annual Partial Nephrectomy Volume in Each of the Six Key Steps [file jamanetwopen-e2421696-s001.pdf]

Supplemental Online Content

Wang Y, Wilder S, Hijazi M, et al; for the Michigan Urological Surgery Improvement Collaborative. Surgeon skill and perioperative outcomes in robot-assisted partial nephrectomy. *JAMA Netw Open.* 2024;7(7):e2421696. doi:10.1001/jamanetworkopen.2024.21696

eFigure 1. Outline of the Video Review Process

eFigure 2. Example of a Score Card Given to Participants

eFigure 3. Survey Sent to Submitters and Reviewers to Evaluate Satisfaction and Efficacy of the Video Review Process

eFigure 4. The MUSIC-KIDNEY Urology YouTube Channel Contains Shortened Videos of All 127 Submitted Video Clips

eTable 1. The Association Between Surgeon Skill Scores and Rate of Risk Adjusted Outcomes (%) in Each of the Six Key Steps

eTable 2. The Association Between Surgeon Skill Scores and Annual Partial Nephrectomy Volume in Each of the Six Key Steps

This supplemental material has been provided by the authors to give readers additional information about their work.

© 2024 Wang Y et al. *JAMA Network Open.*

eFigure 1. Outline of the video review process.

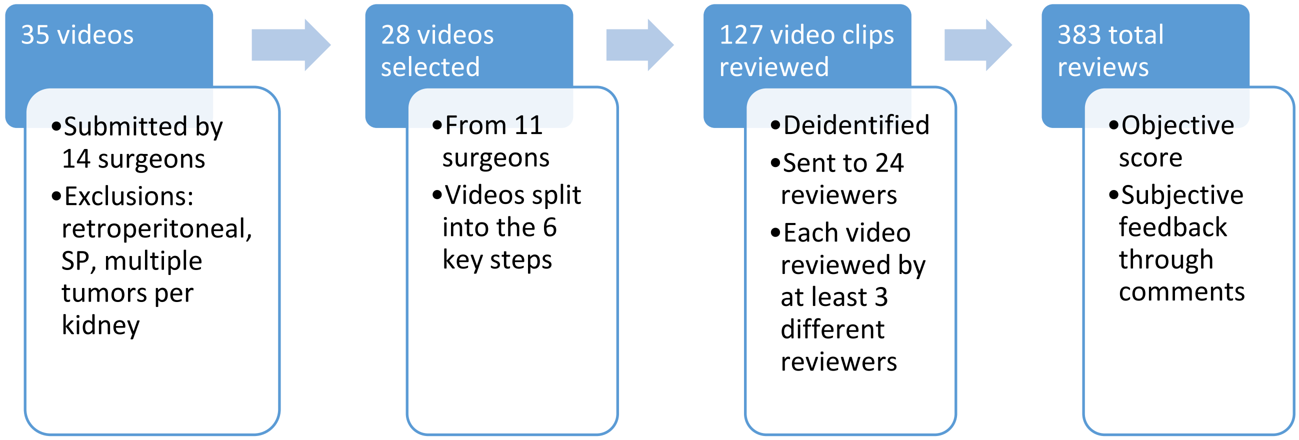

SP = Da Vinci SP robot (Intuitive, Sunnyvale, CA)

© 2024 Wang Y et al. *JAMA Network Open.*

eFigure 2. Example of a score card given to participating surgeons and reviewers. The report in **(A)** includes a graph with one individual surgeon's mean scores compared to all surgeons who submitted videos. **(B)** Includes a link to the submitted video as well as de-identified feedback for each step on a different surgeon's report. Example of comments are shown below. Comments have been paraphrased and shortened somewhat from the complete submitted comments.

(A)

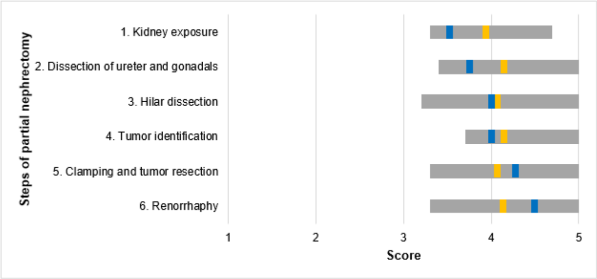

	Minimum	Maximum	Mean (all surgeons)	Your mean
1. Kidney exposure	3.3	4.7	3.9	3.5
2. Dissection of ureter and gonadals	3.4	5	4.2	3.7
3. Hilar dissection	3.2	5	4.1	4.0
4. Tumor identification	3.7	5	4.2	4.0
5. Clamping and tumor resection	3.3	5	4.1	4.3
6. Renorrhaphy	3.3	5	4.2	4.5

Summary:

- You submitted 2 videos.
- Your mean score for all steps was 4.0 (compared to the mean of 4.2 for all submitting surgeons)
- You scored at or above average on (5) clamping and tumor resection and (6) renorrhaphy
- You scored below average on (1) kidney exposure, (2) dissection of ureter and gonadals, (3) hilar dissection, and (4) tumor identification

(B)

Video 1: link to video

**1. Kidney exposure**<br>(Mean score: 3.7)
Reviewer 1: Too much unnecessary dissection, trouble finding the right planes.<br>Reviewer 2: Good exposure.<br>Reviewer 3: Safely able to expose kidney and mobilize bowel.
**2. Dissection of ureter and gonadals**<br>(Mean score: 4.3)
Reviewer 1: There was some teaching during this video. Excellent anatomical exposure.<br>Reviewer 2: Adequate and safe dissection on gonadal and ureter.<br>Reviewer 3: Should have focused less on the dissection posteriorly over psoas muscle.
**3. Hilar dissection**<br>(Mean score: 5)
Reviewer 1: Safe and expeditious; excellent exposure of hilar vessels.<br>Reviewer 2: Great job.<br>Reviewer 3: Excellent hilar exposure and traction exposing renal artery fully.
**4. Tumor identification**<br>(Mean score 4.7)
Reviewer 1: Leave some fat on tumor for margin, could be used as a handle.<br>Reviewer 2: Good visualization of tumor. Good use of ultrasound.<br>Reviewer 3: Nice wide dissection allowed for full visualization of tumor.
**5. Clamping and tumor resection**<br>(Mean score: 5)
Reviewer 1: Clean margins, good control of hemostasis.<br>Reviewer 2: Excellent exposure of hilar vessels.<br>Reviewer 3: Good technique.
**6. Renorrhaphy**<br>(Mean score 4.3)
Reviewer 1: A few clips lead to minor trauma.<br>Reviewer 2: The orientation of the clips was not all parallel to defect, the closure could not be tensioned adequately, leaving gaps between edges of defect. Concern for delayed bleeding.<br>Reviewer 3: This is different from the technique I use, but it is also very nice.

© 2024 Wang Y et al. *JAMA Network Open.*

eFigure 3. Survey sent to submitters and reviewers to evaluate satisfaction and efficacy of the video review process. Survey questions used a Likert scale: 1=*highly disagree*, 2=*disagree*, 3=*neutral*, 4=*agree*, 5=*highly agree*.

Survey for submitters

Please rate your level of agreement with the following statements:

I would consider submission of additional videos in the future.	1	2	3	4	5
I found my specific report, including the written feedback from peer reviewers, to be useful.	1	2	3	4	5
I found the scoring tool to be a useful way of evaluating any technical skills.	1	2	3	4	5
Overall, I found review of my video(s) to be a useful experience.	1	2	3	4	5

What specifically did you find valuable about the process of video review as a video submitter? Please select all that apply:

- ☐ Identified areas of improvement in own technical skill
- ☐ Learned from another surgeon's feedback
- ☐ Utility in the education of trainees
- ☐ Other: ____

Survey for reviewers

Please rate your level of agreement with the following statements:

I would consider participation in future video reviews.	1	2	3	4	5
I found the scoring tool to be a useful way of evaluating technical skill.	1	2	3	4	5
Overall, I found the video review process to be a useful experience.	1	2	3	4	5

What did you specifically find valuable about the process of video review as a reviewer? Please select all that apply:

- ☐ Identified areas of improvement in own technical skill
- ☐ Ability to learn from other surgeons' technique
- ☐ Utility in education of trainees
- ☐ Other: ____

Additional comments: ____

© 2024 Wang Y et al. *JAMA Network Open.*

eFigure 4. The MUSIC-KIDNEY Urology YouTube channel contains shortened videos of all 127 submitted video clips. Each video description contains the corresponding scores given by the reviewers as well as a summary of the written feedback. The link to the YouTube channel is included along with a QR code to the site.
https://www.youtube.com/@MUSICKUrology/playlists

© 2024 Wang Y et al. *JAMA Network Open.*

eTable 1. The association between surgeon skill scores and rate of risk-adjusted outcomes (%) in each of the six key steps: (1) exposure of the kidney, (2) identification of the ureter and gonadal vessels, (3) hilar dissection, (4) tumor localization and exposure, (5) clamping and tumor resection, and (6) renorrhaphy. Bolded p-values are significant.

Outcome	Key step of partial nephrectomy	ß Coefficient	95% CI	p-value
Length of stay	1	3.0%	1.2% – 4.8%	**0.001**
	2	-6.1%	-8.2% – -4.0%	**<0.001**
	3	-1.0%	-2.1% – 0.2%	0.095
	4	-2.3%	-3.6% – -1.0%	**<0.001**
	5	-3.7%	-4.7% – -2.7%	**<0.001**
	6	-1.6%	-3.0% – -0.2%	0.030
Estimated blood loss	1	3.5%	3.0% – 3.9%	**<0.001**
	2	1.2%	0.5% – 1.8%	**<0.001**
	3	-2.1%	-2.4% – -1.8%	**<0.001**
	4	-2.0%	-2.3% – -1.6%	**<0.001**
	5	-1.4%	-1.7% – -1.1%	**<0.001**
	6	-2.4%	-2.8% – -2.1%	**<0.001**
Warm ischemia time	1	-4.0%	-5.3% – -2.7%	**<0.001**
	2	-0.8%	-2.3% – 0.8%	0.3
	3	2.2%	1.4% – 3.0%	**<0.001**
	4	1.0%	0% – 1.9%	0.058
	5	1.4%	0.6% – 2.1%	**<0.001**
	6	3.7%	2.1% – 5.4%	**<0.001**
Positive surgical margins	1	3.4%	2.2% – 4.6%	**<0.001**
	2	-2.0%	-3.4% – -0.5%	0.009
	3	-3.8%	-4.5% – -3.1%	**<0.001**
	4	-1.0%	-1.9% – 0%	0.040
	5	-3.5%	-4.2% – -2.9%	**<0.001**
	6	-5.6%	-6.5% – -4.6%	**<0.001**
30-day emergency department visits	1	2.0%	0.8% – 3.2%	**0.001**
	2	-0.8%	-2.2% – 0.7%	0.3
	3	-0.6%	-1.4% – 0.2%	0.14
	4	-2.9%	-3.7% – -2.0%	**<0.001**
	5	-0.8%	-1.5% – -0.1%	0.026
	6	-3.0%	-3.9% – -2.1%	**<0.001**
30-day readmissions	1	4.2%	2.9% – 5.6%	**<0.001**
	2	-3.4%	-5.1% – -1.7%	**<0.001**
	3	-1.7%	-2.6% – -0.8%	**<0.001**
	4	-4.4%	-5.3% – -3.4%	**<0.001**
	5	-3.1%	-3.8% – -2.3%	**<0.001**
	6	-3.7%	-4.8% – -2.7%	**<0.001**

© 2024 Wang Y et al. *JAMA Network Open.*

eTable 2. The association between surgeon skill scores and annual partial nephrectomy volume in each of the six key steps. Bolded p-values are significant.

**Key step of partial nephrectomy**	**ß Coefficient**	**95% CI**	**p-value**
(1) Exposure of the kidney	-11.2	-12.3 – -10.2	**<0.001**
(2) Identification of the ureter and gonadal vessels	-2.4	-4.2 – -0.5	0.012
(3) Hilar dissection	8.0	7.5 – 8.6	**<0.001**
(4) Tumor localization and exposure	4.8	3.6 – 5.9	**<0.001**
(5) Clamping and tumor resection	4.8	4.0 – 5.7	**<0.001**
(6) Renorrhaphy	5.8	4.5 – 7.1	**<0.001**

© 2024 Wang Y et al. *JAMA Network Open.*
