# Supplementary material for: Surgeon Skill and Perioperative Outcomes in Robot-Assisted Partial Nephrectomy
Source: JAMA Netw Open. 2024 Jul 15;7(7):e2421696. doi: 10.1001/jamanetworkopen.2024.21696 (PMC11250260; doi:10.1001/jamanetworkopen.2024.21696)
Supplement: Supplement 2. — Data Sharing Statement [file jamanetwopen-e2421696-s002.pdf]

Data Sharing Statement

Wang. Surgeon Skill and Perioperative Outcomes in Robot-Assisted Partial Nephrectomy. *JAMA Netw Open*. Published July 12, 2024. doi:10.1001/jamanetworkopen.2024.21696

Data
Data available: No

Additional Information
Explanation for why data not available: Our data are not available publicly. However, we can provide deidentified data if requested.
